# Antidiabetic Properties of *Ficus deltoidea* Jack: A Review of In Vitro, In Vivo, and Clinical Evidence

**DOI:** 10.3390/life16081311

**Published:** 2026-08-10

**Authors:** Siti Hajar Adam, Nor Syaza Syahirah Amat Junaidi, Shariff Halim, Mohd Helmy Mokhtar

**Affiliations:** 1 Pre-Clinical Department, Faculty of Medicine and Defense Health, Universiti Pertahanan Nasional Malaysia, Kuala Lumpur 57000, Malaysia; siti.hajar@upnm.edu.my (S.H.A.); norsyaza@upnm.edu.my (N.S.S.A.J.); 2 Faculty of Health Sciences, Universiti Teknologi MARA Cawangan Pulau Pinang, Kampus Bertam, Kepala Batas 13200, Malaysia; halimshariff@uitm.edu.my; 3 Department of Physiology, Faculty of Medicine, Universiti Kebangsaan Malaysia, Kuala Lumpur 56000, Malaysia

**Keywords:** *Ficus deltoidea*, Mas Cotek, diabetes mellitus, vitexin, isovitexin, α-glucosidase, insulin secretion, PTP1B

## Abstract

*Ficus deltoidea* Jack (Moraceae), locally known as Mas Cotek, is a medicinal plant traditionally used throughout Southeast Asia for the management of diabetes mellitus. This review summarises the available evidence on the antidiabetic properties of *F. deltoidea* based on eleven in vitro, nine in vivo and one clinical study identified through a structured literature search. In vitro investigations show that *F. deltoidea* inhibits α-glucosidase and α-amylase, stimulates insulin secretion in pancreatic β-cells via both K^+^-ATP channel-dependent and -independent pathways, enhances glucose uptake in hepatocytes and adipocytes, promotes adiponectin secretion and inhibits protein tyrosine phosphatase 1B (PTP1B). Vitexin and isovitexin, the predominant C-glycosyl flavonoids in *F. deltoidea* leaves, appear to be the main bioactive compounds responsible for these effects. Meanwhile, in vivo studies in streptozotocin-induced diabetic rodents report dose-dependent reductions in fasting blood glucose, improved glucose tolerance, restoration of pancreatic islet architecture, modulation of hepatic gluconeogenic and glucose-metabolic genes, and protection against diabetic nephropathy and bone loss. Inter-varietal differences in chemical composition and biological activity were observed, with *var. trengganuensis* and *var. intermedia* reported as the most active. The only available clinical trial in adults with prediabetes (1000 mg/day for 8 weeks) showed a reduction in LDL and total cholesterol but no significant change in fasting blood glucose or insulin. The discrepancy between preclinical and clinical findings highlights the need for standardised extracts, pharmacokinetic studies and adequately powered clinical trials in patients with established type 2 diabetes mellitus.

## 1. Introduction

Diabetes mellitus (DM) is a chronic metabolic disorder characterised by persistent hyperglycaemia resulting from defects in insulin secretion, insulin action or both [1,2]. Long-term hyperglycaemia is associated with damage to multiple organs including the eyes, kidneys, nerves, heart and blood vessels [3,4]. According to the International Diabetes Federation, approximately 589 million adults were living with diabetes in 2024, and this number is projected to rise to 853 million by 2050, with the majority of cases concentrated in low- and middle-income countries [5]. Type 2 diabetes mellitus (T2DM) accounts for the majority of these cases.

Current management of T2DM relies on pharmacological agents such as metformin, sulfonylureas, thiazolidinediones, dipeptidyl peptidase-4 inhibitors, glucagon-like peptide-1 receptor agonists and sodium-glucose co-transporter-2 inhibitors [6,7]. Although these drugs are effective at lowering blood glucose, their long-term use is often associated with adverse effects including gastrointestinal disturbances, weight gain, hypoglycaemia, vitamin B12 deficiency and an increased risk of cardiovascular disease [8,9]. These effects are largely drug-specific. Sulfonylureas and thiazolidinediones promote weight gain through insulin-driven lipogenesis and PPAR-γ-mediated adipogenesis, metformin-related gastrointestinal issues arise from altered gut motility and bile acid signalling, and prolonged metformin use impairs vitamin B12 absorption by reducing calcium-dependent uptake in the terminal ileum. Most conventional agents also act on a single molecular target and do not adequately address the multifactorial nature of T2DM, which involves insulin resistance, β-cell dysfunction, oxidative stress and chronic inflammation [10,11].

These limitations have generated growing interest in medicinal plants as complementary therapies for DM [12]. Herbal preparations are often perceived to have a wider safety margin and to act on several pathways involved in glucose homeostasis through their content of polyphenols, flavonoids, terpenes and other secondary metabolites [12,13]. Several Malaysian medicinal plants have been studied for their antidiabetic potential, including *Gynura procumbens*, *Cosmos caudatus*, *Orthosiphon stamineus* and *Piper sarmentosum* [14]. Among these, *Ficus deltoidea*, has been one of the most extensively investigated species, owing to its long history of traditional use and its rich content of compounds with antidiabetic potential.

*Ficus deltoidea* Jack (Moraceae), locally known as Mas Cotek in Malaysia and Pokok Suji in Indonesia, is an epiphytic shrub distributed across Southeast Asia [14]. The leaves, fruits and roots have traditionally been used in the form of decoctions or infusions for the treatment of diabetes, hypertension, gout and post-partum recovery [14]. Phytochemical investigations have shown that *F. deltoidea* is rich in flavonoid C-glycosides, particularly vitexin and isovitexin, as well as phenolic acids, tannins and triterpenes [14,15]. Several preclinical studies have reported that these compounds contribute to glucose-lowering activity through multiple mechanisms, including inhibition of carbohydrate-digesting enzymes, stimulation of insulin secretion, enhancement of glucose uptake and reduction in oxidative stress in the pancreas [16,17].

Despite this expanding body of preclinical evidence, the available data on the antidiabetic activity of *F. deltoidea* have not been comprehensively reviewed in light of recently published studies on its effect on hepatic gluconeogenesis, PTP1B signalling, diabetic nephropathy and bone metabolism. The present narrative review aims to provide an updated synthesis of the in vitro, in vivo and clinical evidence on the antidiabetic activity of *F. deltoidea*, with particular attention to the underlying mechanisms of action, the contribution of vitexin and isovitexin, inter-varietal differences and the translational gap between preclinical and clinical findings.

## 2. Methodology

A structured literature search was conducted using PubMed, Scopus, ScienceDirect and Google Scholar for articles published between January 2000 and June 2026. The search terms included combinations of “*Ficus deltoidea*”, “Mas Cotek”, “vitexin”, “isovitexin”, “diabetes”, “antidiabetic”, “hyperglycaemia”, “insulin”, “glucose uptake”, “α-glucosidase” and “PTP1B”, combined with the Boolean operators AND and OR. Reference lists of retrieved articles and previous reviews were also examined to identify additional studies. Studies were included if they (i) were original research articles published in English, (ii) evaluated extracts, fractions or isolated compounds of *F. deltoidea* in the context of DM or glucose metabolism, and (iii) reported in vitro assays, in vivo experiments in animal models, or human clinical trials. Review articles, conference abstracts, editorials and studies not directly related to antidiabetic activity were excluded. For each included study, information was extracted on the plant part used, extract or compound, dose and duration, experimental model, outcomes measured and main findings. A total of 21 primary studies met the inclusion criteria and were used to construct the narrative synthesis presented in Section 4, Section 5 and Section 6.

## 3. Phytochemistry of *Ficus deltoidea*

*F. deltoidea* contains a range of secondary metabolites that are considered to underlie its pharmacological activity. The leaves are the most extensively studied part of the plant and contain flavonoid C-glycosides (mainly vitexin and isovitexin), flavonols (quercetin, kaempferol and their glycosides), phenolic acids (caffeic acid, gallic acid, chlorogenic acid), proanthocyanidins, tannins and triterpenes [14,15]. The qualitative and quantitative composition of these compounds varies among the seven recognised botanical varieties (*var. deltoidea*, *var. angustifolia*, *var. trengganuensis*, *var. intermedia*, *var. kunstleri*, *var. motleyana* and *var. bilobata*) and is also influenced by plant maturity, growing conditions, harvesting season, extraction solvent and analytical method [14,18].

### 3.1. Flavonoid C-Glycosides: Vitexin and Isovitexin

Vitexin (apigenin-8-C-glucoside) and isovitexin (apigenin-6-C-glucoside) are the major C-glycosyl flavonoids identified in *F. deltoidea* leaves and are commonly used as chemical markers for quality control of standardised extracts [19,20]. HPLC analyses of methanolic leaf extracts have reported vitexin and isovitexin contents in the range of approximately 2.8–4.2% w/w and 1.5–2.8% w/w respectively, although values vary considerably between varieties and studies [21]. The reported content of both compounds is also strongly solvent-dependent, with hydroalcoholic extraction generally yielding the highest recovery of these C-glycosides, as discussed in detail in Section 3.3. Varietal comparisons using standardised extracts have further shown quantitative differences in insulinotropic and hypoglycaemic potency between *F. deltoidea* varieties (e.g., *var. trengganuensis*, *var. intermedia*, *var. kunstleri*) [18,22]. This suggests that inter-varietal chemical variation, potentially including vitexin and isovitexin content, contributes to the differences in bioactivity described in Section 4 and Section 5. However, dedicated comparative phytochemical profiling across varieties remains limited.

Compared with the more common O-glycosylated flavonoids, C-glycosides are relatively resistant to hydrolysis by intestinal glucosidases, which may contribute to their stability during gastrointestinal transit [19,20,23]. Vitexin and isovitexin have been reported to inhibit α-glucosidase, scavenge free radicals and protect β-cells against oxidative damage, supporting their role as the main bioactive markers of *F. deltoidea* [23,24].

### 3.2. Polyphenolic and Triterpene Compounds

**Figure 1:**
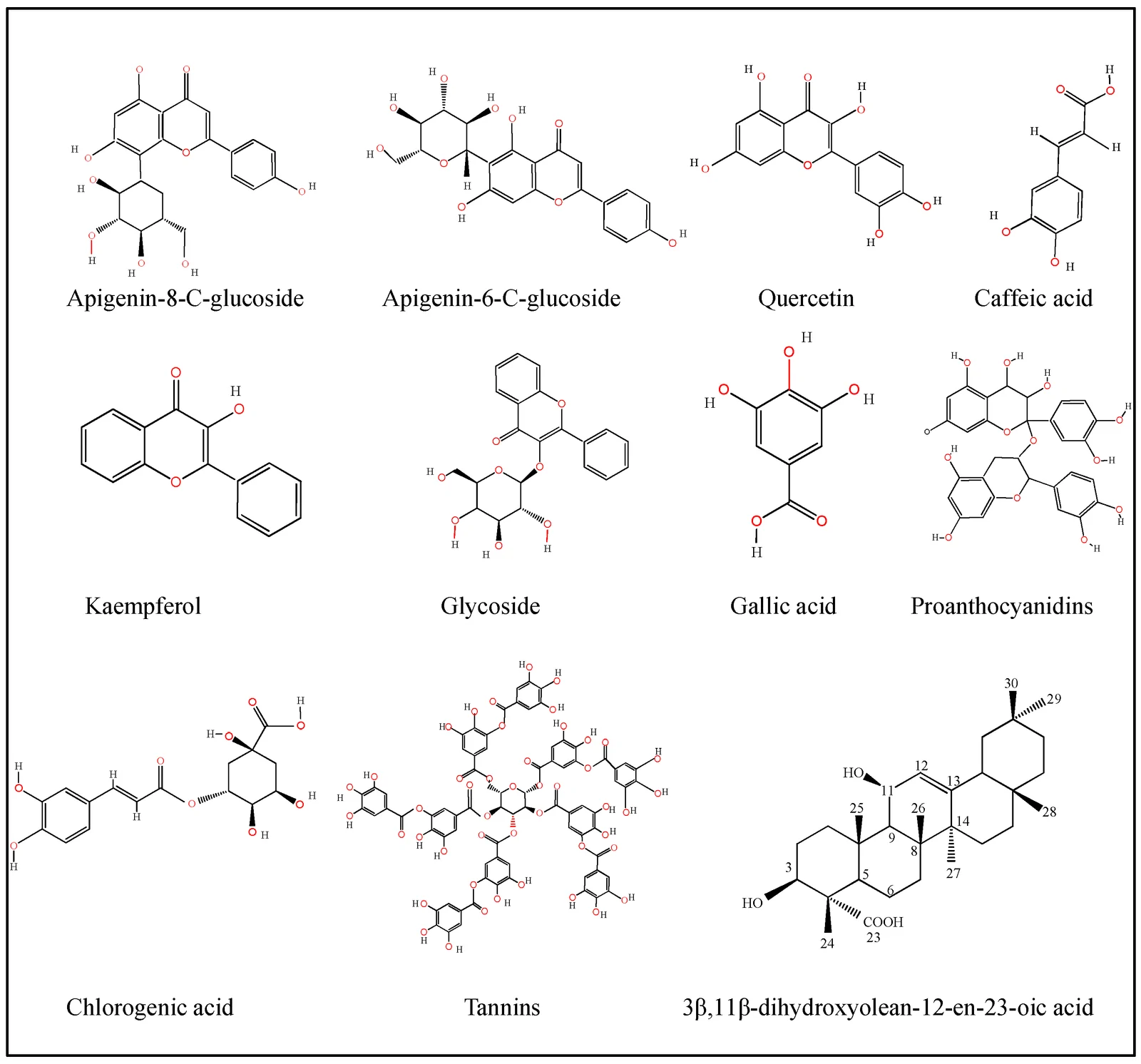
Chemical structures of selected phytochemicals reported in *Ficus deltoidea*.

In addition to flavonoids, *F. deltoidea* contains phenolic acids and triterpenes that have been associated with its antidiabetic and antioxidant activity. Caffeic acid, gallic acid and proanthocyanidins contribute to the scavenging of reactive oxygen species and the attenuation of oxidative stress in hyperglycaemic states [14,23]. A triterpene fraction containing 3β,11β-dihydroxyolean-12-en-23-oic acid, isolated from a 70% ethanol extract, has been reported to inhibit PTP1B in vitro and to be enriched in fractions with strong antidiabetic activity in vivo [25]. The relative contribution of individual flavonoids, phenolic acids and triterpenes to the overall pharmacological activity of *F. deltoidea* remains incompletely characterised and is likely to depend on extract composition. The chemical structures of selected phytochemicals reported in *F. deltoidea* are shown in Figure 1.

### 3.3. Extract Preparation and Standardisation

The pharmacological activity of *F. deltoidea* extracts is strongly influenced by extract preparation. Aqueous extracts preferentially recover flavonoid C-glycosides such as vitexin and isovitexin, as well as phenolic acids, whereas ethanolic and methanolic extracts additionally recover fewer polar constituents including flavonols and triterpenes. Hydroalcoholic extracts (typically 50–70% ethanol in water) have been reported to yield the highest contents of vitexin and isovitexin, reflecting the intermediate polarity of these amphipathic compounds. Aqueous extracts more closely reflect the traditional preparation of *F. deltoidea* as a decoction or tea, whereas ethanolic and methanolic extracts are more commonly used in pharmacological screening.

Reproducibility across studies is further affected by plant maturity at harvest, geographical origin, harvesting season and post-harvest handling, all of which influence the qualitative and quantitative composition of the extract. Few of the studies reviewed quantified vitexin and isovitexin contents in the extracts used, which precludes direct comparison of pharmacological activity on a per-marker-compound basis. Standardisation to a defined vitexin and isovitexin content represents a necessary step to advance *F. deltoidea* research towards clinical translation.

## 4. Antidiabetic Properties of *Ficus deltoidea*: Evidence from In Vitro Studies

In vitro studies on *F. deltoidea* have examined three main aspects of antidiabetic activity: inhibition of carbohydrate-digesting enzymes, stimulation of insulin secretion in pancreatic β-cell lines, and enhancement of glucose uptake in hepatocytes and adipocytes. More recent investigations have expanded these observations to include adiponectin secretion, PTP1B inhibition, endothelial protection and effects in cellular models of non-alcoholic fatty liver disease and neuroinflammation.

### 4.1. Inhibition of α-Glucosidase and α-Amylase

Inhibition of intestinal α-glucosidase and α-amylase delays the digestion of complex carbohydrates and reduces postprandial hyperglycaemia, an approach exemplified by acarbose. These enzymes are essential for carbohydrate metabolism, with α-amylase facilitating the breakdown of starch into smaller oligosaccharides, and α-glucosidase subsequently converting these products into absorbable monosaccharides [26]. Inhibition of either enzyme can limit glucose absorption from the gastrointestinal tract and reduce postprandial blood glucose rises, which contribute to poor glycaemic control in people with T2DM.

Aqueous and ethyl acetate fractions of *F. deltoidea* fruits have been reported to inhibit α-glucosidase but to exert little effect on α-amylase, with the aqueous fractions of *var. angustifolia* and *var. kunstleri* showing the strongest inhibition in a dose-dependent manner [27]. In contrast, Abu Bakar et al. reported that a 50% ethanol-water extract of *F. deltoidea* leaves inhibited α-amylase in a concentration-dependent manner, with molecular docking suggesting that vitexin and isovitexin bound to the active site of the enzyme [28]. The difference between these two studies likely reflects variation in plant part (fruit versus leaf), extraction solvent and the predominant phytochemical class in each preparation. The leaves are enriched in vitexin and isovitexin, whereas the fruits contain a different balance of flavonoid C-glycosides and proanthocyanidins [14,20].

Studies on isolated compounds have shown that vitexin is a more potent α-glucosidase inhibitor than isovitexin, with IC_50_ values in the low micromolar range [19,20,24]. He et al. reported that vitexin acts as a mixed-type or uncompetitive inhibitor of α-glucosidase rather than as a purely competitive inhibitor like acarbose. This suggests that combinations of vitexin and acarbose may produce additive or synergistic inhibition of the enzyme [20]. Unlike competitive inhibitors, which bind only to the active site, mixed-type and uncompetitive inhibitors may interact with enzyme-substrate complexes or allosteric sites, potentially resulting in sustained inhibitory effects even at high substrate concentrations [29]. This characteristic may offer therapeutic benefits in regulating postprandial glucose levels across varying dietary carbohydrate intakes.

Published mechanistic studies indicate that vitexin acts as an uncompetitive inhibitor of α-glucosidase, with a reported IC_50_ of approximately 52.8 μM, and a potency greater than that of acarbose in the same assay [20]. Notably, vitexin has been shown to act synergistically with acarbose rather than through a simple additive effect, which is consistent with its non-active-site binding mode [20]. For α-amylase, the reported mode of inhibition by *F. deltoidea* extracts varies from competitive to mixed-type across studies, suggesting that inhibition by crude extracts reflects the combined contribution of flavonoid C-glycosides and other constituents such as tannins and proanthocyanidins [27,28].

These findings support a role for *F. deltoidea* leaf extracts in limiting postprandial glucose excursions, although the relative contributions of vitexin, isovitexin and other phenolic constituents to the total inhibitory activity have not yet been fully elucidated. However, most available data derive from in vitro enzyme assays and computational studies, which may not adequately reflect the complexity of gastrointestinal digestion and absorption in vivo. Therefore, future research should prioritise the bioavailability, intestinal stability, and pharmacokinetic behaviour of these compounds, as well as their effectiveness in animal models and human studies. Such investigations would help clarify the translational relevance of α-glucosidase and α-amylase inhibition by *F. deltoidea* and its potential as a complementary approach to managing postprandial hyperglycaemia.

### 4.2. Stimulation of Insulin Secretion

The BRIN-BD11 cell line is widely used as an experimental model for assessing insulin secretagogues because it retains many functional characteristics of pancreatic β-cells, including glucose responsiveness and regulated insulin secretion [30]. It has been reported that aqueous, ethanolic and methanolic extracts of *F. deltoidea* stimulate insulin secretion in BRIN-BD11 pancreatic β-cells in a dose-dependent manner [31]. The ability of *F. deltoidea* extracts to enhance insulin secretion suggests that the plant may contain bioactive constituents capable of directly modulating β-cell function and increasing insulin availability under hyperglycaemic conditions. Mechanistic studies indicate that the insulinotropic effect is mediated by both K^+^-ATP channel-dependent and -independent pathways and is accompanied by mobilisation of intracellular calcium stores. The K^+^-ATP channel-dependent pathway, which involves channel closure, membrane depolarisation and opening of voltage-dependent calcium channels, is also the target of sulfonylureas [32]. The additional involvement of intracellular calcium stores and channel-independent pathways may help sustain insulin secretion when the conventional pathway is impaired. Mobilisation of intracellular calcium stores, especially from the endoplasmic reticulum, can enhance the secretory response and improve insulin release without reliance on external calcium influx [33,34].

Yahaya et al. compared standardised methanolic extracts of seven *F. deltoidea* varieties in BRIN-BD11 cells and reported that all varieties stimulated insulin secretion, but only *F. deltoidea var. trengganuensis* acted predominantly through a K^+^-ATP-independent pathway [18]. The other varieties, including *var. deltoidea* and *var. angustifolia*, showed dose-dependent K^+^-ATP-dependent secretion. This inter-varietal variation in mechanism is consistent with differences in the chemical composition of the varieties and indicates that variety selection may influence the pharmacological profile of *F. deltoidea* preparations. The results highlight the importance of botanical and phytochemical standardisation when evaluating the antidiabetic efficacy of *F. deltoidea*. Although all varieties are classified under the same species, significant differences have been observed in the concentrations of flavonoids, phenolic acids, proanthocyanidins, and other secondary metabolites among distinct types [35]. These changes may alter the relative abundance of bioactive molecules that mediate insulinotropic activity, thereby affecting the fundamental mechanisms of action.

### 4.3. Enhancement of Glucose Uptake

Adam et al. (2009) showed that several extracts and fractions of *F. deltoidea* enhanced both basal and insulin-stimulated glucose uptake in the Chang liver cell line, with ethanolic, methanolic and acidified or basified fractions showing both insulin-mimetic and insulin-sensitising activity [36]. Insulin-mimetic activity refers to a compound’s ability to promote glucose uptake independently of insulin, while insulin-sensitising activity improves cellular responsiveness to both endogenous and exogenous insulin [37,38]. The presence of both activities suggests that *F. deltoidea* may influence multiple aspects of glucose homeostasis and could improve glycaemic control through complementary mechanisms.

Adipocytes play a crucial role in systemic glucose regulation and energy metabolism, accounting for a significant proportion of insulin-mediated glucose disposal [39]. In a subsequent study using 3T3-F442A adipocytes, methanolic and ethanolic extracts increased both basal and insulin-mediated glucose uptake in a concentration-dependent manner [31]. The improvement in glucose uptake without an increase in GLUT4 mRNA expression suggests that *F. deltoidea* promotes the translocation of pre-existing transporters to the cell membrane rather than increasing their synthesis [31,36]. Under physiological conditions, insulin enhances the translocation of intracellular vesicles carrying GLUT4 to the plasma membrane, thereby increasing glucose entry into cells [40]. Furthermore, enhanced glucose disposal in hepatocytes and adipocytes is expected to contribute to lower blood glucose levels and improved insulin sensitivity in vivo [41].

### 4.4. Adiponectin Secretion and PTP1B Inhibition

Adam et al. (2012) further reported that aqueous and methanolic extracts of *F. deltoidea* increased adiponectin secretion from 3T3-F442A adipocytes in a concentration-dependent manner, with up to a 2.46-fold increase at 1000 μg/mL [31]. Adiponectin is an adipokine that improves insulin sensitivity through activation of AMP-activated protein kinase (AMPK) and PPARα and has been shown to be reduced in obesity and T2DM [42]. Stimulation of adiponectin secretion therefore represents an additional, indirect mechanism by which *F. deltoidea* may improve insulin sensitivity.

A previous study conducted bioassay-guided fractionation of a 70% ethanol extract of *F. deltoidea* and identified a triterpene fraction containing 3β,11β-dihydroxyolean-12-en-23-oic acid that inhibited PTP1B by approximately 92% at 200 μg/mL [25]. PTP1B dephosphorylates the activated insulin receptor and insulin receptor substrate-1, thereby attenuating insulin signalling [43]. PTP1B, a key negative regulator of insulin action, has been identified as a potential therapeutic target for improving insulin sensitivity and reducing insulin resistance [44,45]. The significant inhibitory activity observed in this triterpene fraction suggests that compounds other than the commonly studied flavonoids, vitexin and isovitexin, may also contribute to the antidiabetic effects of *F. deltoidea*. However, further research is required to determine the relative contribution of these triterpenes to the plant’s overall pharmacological effects and to elucidate their mechanisms of action in vivo.

### 4.5. Other In Vitro Effects

More recent in vitro studies have examined the effects of *F. deltoidea* in cellular models relevant to diabetic complications. Cardiovascular disease remains a leading cause of morbidity and mortality in individuals with diabetes, as persistent hyperglycaemia, oxidative stress, and inflammation result in endothelial dysfunction and accelerated atherosclerosis [46,47]. Endothelial activation is characterised by increased expression of adhesion molecules, including intercellular adhesion molecule-1 (ICAM-1) and vascular cell adhesion molecule-1 (VCAM-1), which facilitate the recruitment and adhesion of circulating monocytes to the vascular endothelium, a key early event in atherogenesis [48,49]. One study reported that aqueous ethanolic extracts of four *F. deltoidea* varieties (*var. trengganuensis*, *var. kunstleri*, *var. deltoidea* and *var. intermedia*) reduced LPS-induced expression of ICAM-1 and VCAM-1, decreased monocyte–endothelial adhesion and increased eNOS expression in human coronary artery endothelial cells, suggesting an anti-atherogenic effect that may be relevant to diabetes-associated vascular dysfunction [50]. It has been suggested that *F. deltoidea* may interfere with key cellular processes involved in the initiation and progression of atherosclerotic lesions.

Meanwhile, Md Lazi et al. reported that an aqueous crude extract of *F. deltoidea* leaves showed a high total phenolic content (108.2 mg GAE/g), strong DPPH radical scavenging activity (IC_50_ 5.47 μg/mL), and 76% inhibition of lipid peroxidation, with greater activity than the water or ethyl acetate fractions [51]. In the same study, the crude extract suppressed conjugated diene and TBARS formation in copper-induced human LDL oxidation, and inhibited HMG-CoA reductase activity by approximately 88% at 10 mg/mL, comparable to atorvastatin. The oxidative modification of LDL is a key event in the initiation and progression of atherosclerosis, as oxidised LDL promotes endothelial dysfunction, inflammatory responses, and foam cell formation in the arterial wall [52,53]. In addition, as HMG-CoA reductase is the rate-limiting enzyme in cholesterol biosynthesis, its inhibition may contribute to improved lipid homeostasis and reduced hypercholesterolaemia [54]. Furthermore, the crude extract reduced palmitic acid-induced intracellular lipid accumulation in HepG2 cells in a dose-dependent manner in both pre- and post-treatment protocols. This indicates that *F. deltoidea* may positively affect hepatic lipid metabolism, potentially contributing to improved metabolic health as well as its glucose-lowering properties.

Consistent observations were reported by Abrahim et al. in palmitic acid-induced WRL68 human liver cells, where aqueous extracts of three *F. deltoidea* varieties (*var. angustifolia*, *var. trengganuensis* and *var. kunstleri*) reduced lipid accumulation, with *var. kunstleri* showing the strongest effect at 200 μg/mL and altering the expression of proteins involved in oxidative stress regulation and the ubiquitin–proteasome system. Although these studies were not conducted in diabetic models, hepatic steatosis, dyslipidaemia and atherosclerosis frequently coexist with T2DM. The reduction in LDL oxidation and inhibition of HMG-CoA reductase reported here suggest a potential role for *F. deltoidea* in mitigating cardiovascular and hepatic complications associated with the diabetic state.

**Figure 2:**
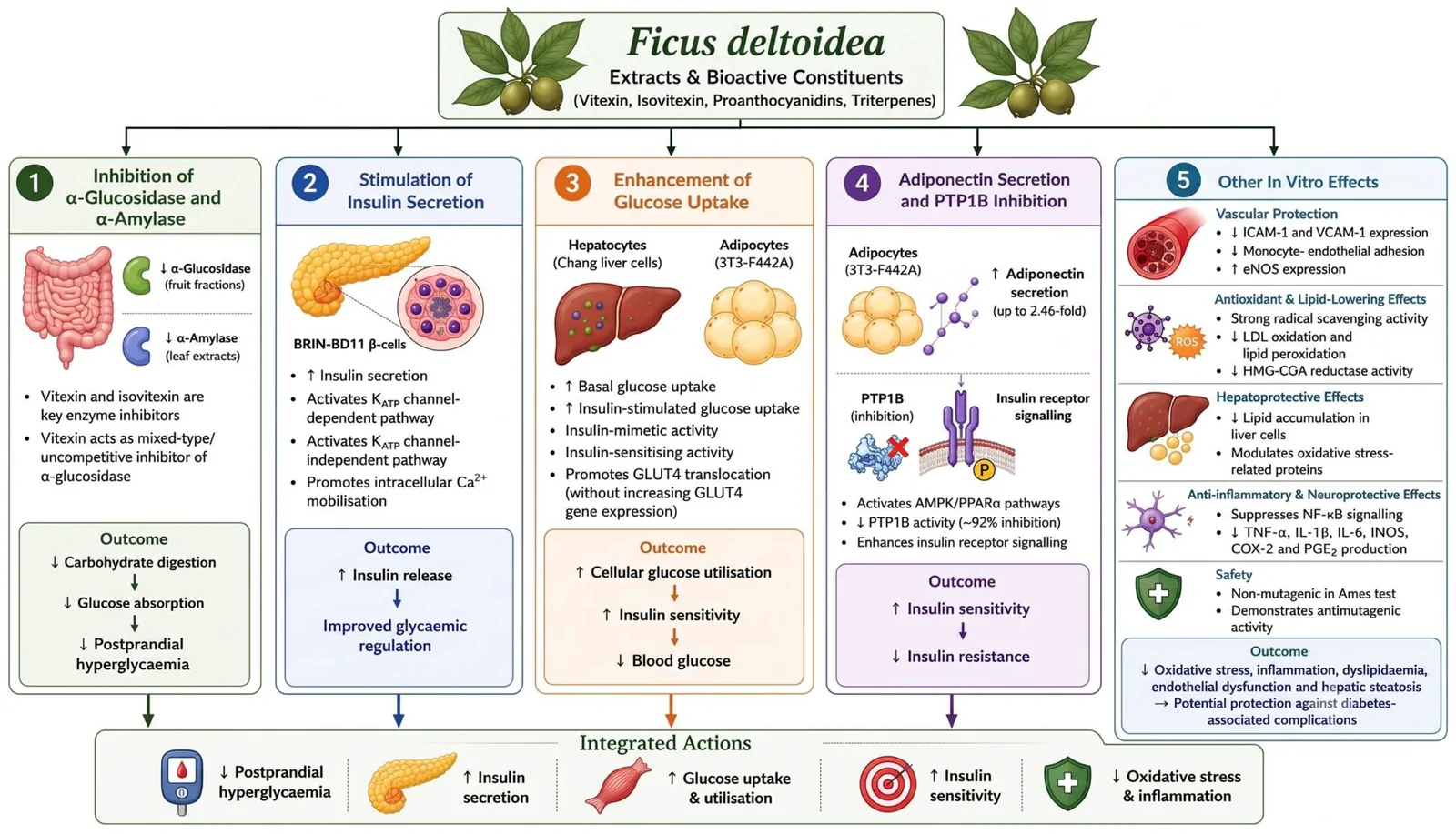
Summary of in vitro antidiabetic mechanisms of *F. deltoidea*. This diagram shows how *F. deltoidea* extracts and their bioactive compounds act at the cellular level to manage diabetes. ↑ indicates increase; ↓ indicates decrease.

A previous study showed that a methanolic extract of *F. deltoidea* attenuated lipopolysaccharide-induced production of pro-inflammatory mediators (TNF-α, IL-1β, IL-6, iNOS, COX-2 and PGE_2_) in BV2 microglial cells, an effect attributed to suppression of the NF-κB signalling pathway [55]. The ability of *F. deltoidea* to block the NF-κB pathway, which plays a key role in chronic inflammation and oxidative stress, suggests it may have anti-inflammatory properties beyond its metabolic effects [56]. Chronic hyperglycaemia has been shown to cause oxidative stress and inflammatory signalling in the nervous system, resulting in neuronal dysfunction and gradual tissue destruction [57]. Together with the antioxidant and anti-inflammatory properties of vitexin and isovitexin, these findings suggest possible neuroprotective effects of *F. deltoidea* that may be relevant to diabetic neuropathy and other neuroinflammatory complications of diabetes. In addition, an aqueous extract of *F. deltoidea var. deltoidea* was not mutagenic in the Ames test using *Salmonella typhimurium* TA 98 and TA 100 strains at concentrations up to 50 mg/mL, and also reduced the revertant colony count induced by 2-aminoanthracene in the presence of metabolic activation, suggesting antimutagenic activity [58]. The extract also attenuated menadione-induced oxidative stress in V79 mouse lung fibroblast cells and showed concentration-dependent ferric-reducing antioxidant power, providing preliminary support for the safety and antioxidant activity of *F. deltoidea* extracts. Table 1 summarises the findings from in vitro studies on *F. deltoidea*, and Figure 2 illustrates its overall antidiabetic potential.

Analysis of the aforementioned in vitro evidence requires consideration of several methodological constraints. Most studies used a single extract preparation without independent replication, and only a small fraction quantified vitexin, isovitexin, or other marker compounds in the tested extract. Botanical verification ranged from formal herbarium submission to casual identification based on morphological characteristics. The cell lines used were diverse (3T3-L1, HepG2, BRIN-BD11, HIT-T15, primary hepatocytes, HCAEC, BV2, V79, WRL68), which limits direct potency comparisons between studies. Dose–response information was reported for numerous endpoints; however, IC50 values were often derived from a restricted dose range. These constraints must be taken into account when comparing results across different investigations.

## 5. Antidiabetic Properties of *Ficus deltoidea*: Evidence from In Vivo Studies

In vivo studies on *F. deltoidea* have used normoglycaemic rodents, streptozotocin (STZ)-induced type 1 DM (T1DM) models, and STZ–nicotinamide (STZ-NAM)-induced T2DM models. Commonly reported endpoints include fasting blood glucose, oral glucose tolerance, serum insulin, lipid profile, hepatic and pancreatic gene expression, pancreatic histology, and markers of oxidative stress. Recent studies have also examined diabetic complications, including nephropathy and bone loss.

### 5.1. Effects on Glucose Tolerance and Fasting Glycaemia

Several animal studies have provided evidence of the antidiabetic efficacy of *F. deltoidea*. In a preliminary study, post-treatment with an aqueous extract of *F. deltoidea* at 50 mg/kg reduced the peak blood glucose response in an oral glucose tolerance test (OGTT) in ICR mice at 180 min post-load [60]. Adam et al. (2010) subsequently reported that an ethanolic extract of *F. deltoidea* reduced both fasting and postprandial hyperglycaemia in STZ-induced diabetic rats, with the lowest tested dose (100 mg/kg) significantly improving glucose tolerance [61]. Noor et al. compared five varieties (*var. trengganuensis*, *var. intermedia*, *var. kunstleri*, *var. deltoidea* and *var. angustifolia*) in normoglycaemic rats and reported that the hypoglycaemic effect was most pronounced with *var. trengganuensis* at 250 mg/kg and *var. intermedia* at 500 mg/kg, with OGTT showing the greatest reduction in plasma glucose at 30 min for *var. intermedia*. These findings indicate that genetic or phytochemical variations among *F. deltoidea* varieties may affect their antihyperglycaemic activity, underscoring the need for plant standardisation and varietal selection in future pharmacological and clinical research. In addition, acute toxicity testing in this study indicated that the median lethal dose (LD_50_) of all varieties exceeded 2000 mg/kg, with no signs of morbidity or mortality [22]. However, acute toxicity data alone are insufficient to determine long-term safety; therefore, additional subacute, chronic, reproductive, and clinical safety studies are necessary before definitive conclusions about the safety profile of *F. deltoidea* can be drawn.

In addition, Ilyanie et al. reported that both methanol and butanol extracts reduced blood glucose in normoglycaemic mice, but only the methanol extract lowered blood glucose in STZ-induced diabetic rats [62]. This observation may be attributed to the presence of insulin-sensitising compounds in the methanol extract in addition to the insulinotropic constituents enriched in the butanol fraction [62]. Taken together, these studies indicate that the antidiabetic activity of *F. deltoidea* is detectable across different diabetes models, although the magnitude of the response is influenced by the underlying pathophysiology of the diabetes model used, the botanical variety, phytochemical profile, and extraction solvent.

### 5.2. Modulation of Hepatic Gluconeogenic and Glucose-Metabolic Genes

Farsi et al. examined the effect of a standardised methanolic extract of *F. deltoidea* (1000 mg/kg) and isolated vitexin and isovitexin (250 and 500 mg/kg) administered for 14 days to STZ-induced diabetic rats [21]. HPLC analysis confirmed high vitexin and isovitexin content in the methanolic extract. Treatment reduced fasting blood glucose, improved glucose tolerance and increased serum insulin, accompanied by downregulation of the hepatic gluconeogenic genes *PEPCK* and *G6Pase* and upregulation of *GK* and *PPARγ* in the liver, as well as *GLUT4* in skeletal muscle. Downregulation of these genes indicates that *F. deltoidea* reduces hepatic glucose production, thereby lowering fasting hyperglycaemia. This pathway is particularly important, as increased gluconeogenesis contributes significantly to elevated blood glucose levels in both T1DM and T2DM [63]. In addition, the pattern of gene expression changes resembles that observed with metformin, particularly the suppression of hepatic glucose production, but with the additional upregulation of GK and PPARγ, which may further enhance hepatic glucose utilisation and adipose tissue function [64].

Another study extended these findings to an STZ-NAM T2DM rat model [25]. A 70% ethanol extract administered at 125–500 mg/kg for 28 days reduced fasting blood glucose, increased serum insulin and improved the lipid profile by decreasing total cholesterol and LDL while normalising HDL. These effects were comparable to those of metformin at 150 mg/kg. At the molecular level, the extract downregulated hepatic *PTP1B* mRNA, suppressed *PEPCK* and *G6Pase* expression and increased *Slc2a2* (GLUT2) and insulin receptor mRNA. The extract significantly reduced hepatic PTP1B mRNA, a negative regulator of insulin signalling, potentially improving insulin sensitivity [65]. It also inhibited the gluconeogenic enzymes PEPCK and G6Pase, thereby decreasing hepatic glucose synthesis and endogenous glucose release [66]. Additionally, the treatment increased *Slc2a2* (GLUT2) expression, which may enhance hepatic glucose transport, sensing, and homeostasis [67]. The extract also restored insulin receptor expression, possibly increasing hepatic sensitivity to circulating insulin. These findings therefore indicate that *F. deltoidea* can attenuate hepatic insulin resistance both upstream via PTP1B inhibition and restoration of insulin receptor expression, and downstream, via suppression of gluconeogenic gene expression.

### 5.3. Pancreatic Protection and β-Cell Preservation

A previous study reported that administering *F. deltoidea* methanolic extract (1000 mg/kg) to STZ-induced diabetic rats for 8 weeks restored the histoarchitecture of pancreatic islets, increased islet size and density and improved insulin secretion [68]. Treatment was associated with increased pancreatic antioxidant enzymes (SOD, GPx) and reduced lipid peroxidation, suggesting that the protective effect on β-cells was mediated, at least in part, by attenuation of oxidative stress. Interestingly, isolated vitexin increased antioxidant enzyme activity but did not reproduce the full insulinotropic effect of the extract, indicating that compounds other than vitexin, possibly isovitexin and triterpenes, also contribute to β-cell function recovery [68].

Consistent findings were reported by Abdel-Rahman et al. in an STZ-NAM T2DM model, in which *F. deltoidea* preserved islet architecture and increased insulin secretion [25]. Vitexin and isovitexin have been reported to protect β-cells from oxidative damage in earlier studies, which is consistent with the antioxidant capacity of these flavonoids [23,24]. Given that oxidative stress significantly contributes to β-cell dysfunction and loss in diabetes, the antioxidant properties of these flavonoids may help preserve β-cell viability, maintain endogenous insulin production, and improve overall glycaemic control.

### 5.4. Protection Against Diabetic Complications

Recent studies have examined the effects of *F. deltoidea* on diabetic complications. An 8-week administration of a standardised methanolic extract of *F. deltoidea* (1000 mg/kg) in STZ-induced diabetic rats has been reported to reduce serum creatinine, blood urea nitrogen and uric acid, preserve glomerular and tubular architecture and reduce renal apoptosis markers (caspase-3, cytochrome c) [69]. These changes were accompanied by increased renal antioxidant enzymes (SOD, CAT, GPx) and reduced oxidative stress markers, suggesting that nephroprotection occurs through mechanisms that are partly independent of glucose lowering. In a separate study, the same group reported that *F. deltoidea* improved bone mineral density and osteocalcin levels in STZ-induced diabetic rats, indicating a beneficial effect on diabetic bone loss [70]. These findings extend the potential therapeutic relevance of *F. deltoidea* beyond glycaemic control to the prevention of diabetic end-organ damage.

**Figure 3:**
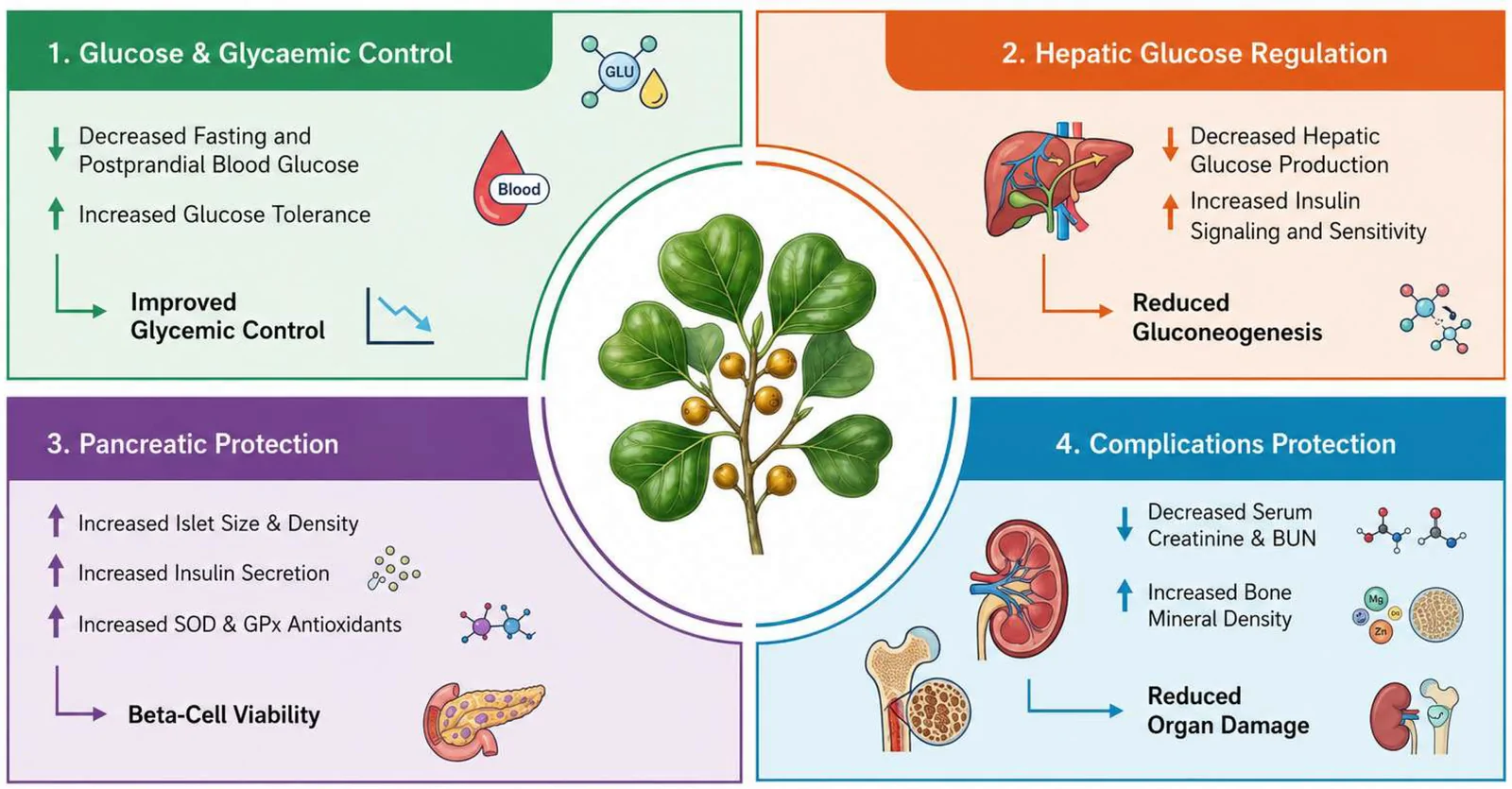
Summary of in vivo antidiabetic mechanisms and organ-protective effects of *F. deltoidea.* This diagram illustrates the key physiological pathways through which *F. deltoidea* exerts its therapeutic effects in animal models. Abbreviation: BUN: Blood Urea Nitrogen; SOD: Superoxide Dismutase; GPx: Glutathione Peroxidase. ↑ indicates increase; ↓ indicates decrease.

The in vivo evidence has several methodological limitations. Sample sizes were generally small (typically 5–8 animals per group). Most studies used STZ or STZ–nicotinamide-induced diabetic rats, with only a small number using high-fat-diet or genetic models that more closely reflect the pathophysiology of human T2DM, and findings were rarely replicated in a second animal model. Extract preparation, dose and treatment duration varied considerably between studies, and only a minority reported the vitexin and isovitexin content of the extract used. Outcome measures were similarly heterogeneous: some studies reported only fasting blood glucose, whereas others assessed glucose tolerance, insulin sensitivity indices, gene expression and histology. Taken together, these factors preclude direct quantitative comparison of efficacy across studies. Table 2 summarises findings from in vivo studies on *F. deltoidea*, and Figure 3 illustrates its overall antidiabetic potential.

## 6. Antidiabetic Properties of *Ficus deltoidea*: Evidence from Clinical Trials

Clinical evidence on *F. deltoidea* is limited to a single randomised controlled trial. Kalman et al. conducted an eight-week, prospective, randomised, double-blind, parallel-group study in 30 adults with prediabetes (fasting blood glucose 100–125 mg/dL), who were randomised to receive *Elaeis guineensis* leaf extract 500 mg/day, *E. guineensis* 1000 mg/day, or *F. deltoidea* leaf extract 1000 mg/day [71]. In the *F. deltoidea* group, total cholesterol and LDL cholesterol were reduced, indicating a potential cardiovascular benefit, but fasting plasma glucose and fasting plasma insulin showed no statistically significant changes. A small, non-significant increase in body weight was observed, and no serious adverse events were reported during the study.

The absence of a significant glycaemic effect contrasts with robust preclinical evidence and warrants careful interpretation. Several methodological considerations may explain this discrepancy. First, the extract used was not standardised to a defined vitexin or isovitexin content, and the bioavailability of the active constituents in humans has not been characterised. Second, the dose of 1000 mg/day was selected empirically without prior human dose-finding studies. Third, the participants were prediabetic with preserved β-cell function and only modest hyperglycaemia, whereas preclinical models typically involve more severe hyperglycaemia. Fourth, the eight-week intervention may have been too short for the transcriptional adaptations observed in animals to translate into measurable changes in fasting glucose. Fifth, the small sample size (n = 10 per group) limited the statistical power to detect modest effects. Adequately powered trials using standardised extracts in patients with established T2DM, longer treatment durations and appropriate biomarkers such as HbA1c are needed before any firm conclusions on clinical efficacy can be drawn.

This limited and equivocal clinical response contrasts with the more consistent glycaemic benefits reported for other botanical antidiabetic agents evaluated in considerably larger and more rigorous clinical programmes. Berberine has shown reproducible reductions in fasting glucose and HbA1c across dozens of RCTs in patients with established T2DM, supported by its dual AMPK-activating and α-glucosidase-inhibiting mechanisms and by well-standardised extracts [72]. *Momordica charantia* and *Gymnema sylvestre* have similarly demonstrated glucose-lowering effects in meta-analyses pooling 10–25 trials, most of which enrolled patients with overt hyperglycaemia rather than prediabetes, where treatment effects are more readily detected [73,74]. Cinnamon trials, though heterogeneous, have likewise benefited from larger cohorts and longer intervention periods than the eight-week window used for *F. deltoidea* [75]. This disparity in trial scale and design, rather than an absence of pharmacological activity, likely explains *F. deltoidea’s* inconclusive glycaemic outcomes, and it also underlies why only one clinical trial exists for this species: unlike berberine or cinnamon, which are globally marketed and commercially standardised, *F. deltoidea* remains a regionally used plant confined largely to Malaysian and Indonesian traditional medicine, limiting the funding available for large trials; it also lacks a validated, marker-compound-standardised extract needed for regulatory approval for human testing, and research has so far been concentrated in academic rather than industry-funded settings. Table 3 summarises findings from clinical trial on *F. deltoidea*.

## 7. Discussion

### 7.1. Pharmacokinetics, Bioavailability and Translational Considerations

A key barrier to the clinical translation of *F. deltoidea* is the limited pharmacokinetic characterisation of its main bioactive constituents. Vitexin and isovitexin are C-glycosyl flavonoids that resist hydrolysis by intestinal α-glucosidases yet show low oral bioavailability in rodents, with an intestinal first-pass extraction of approximately 94% and peak plasma concentrations in the submicromolar range after typical oral doses. Both compounds undergo phase II metabolism through glucuronidation, sulfation and methylation, and the C-glycosyl bond can be cleaved by intestinal microbiota to release the aglycone apigenin, which has distinct pharmacokinetic and pharmacological properties [20,76]. The plasma concentrations achieved after oral administration are therefore likely to be well below the concentrations required for α-glucosidase inhibition in vitro and part of the pharmacological activity observed in preclinical models may reflect the contribution of metabolites rather than vitexin itself [20].

The rodent doses used in preclinical studies (typically 100–1000 mg/kg in rats) translate, using body-surface-area allometric scaling, to human equivalent doses of approximately 16–160 mg/kg, or about 1100–11 000 mg for a 70 kg adult [77]. The single completed clinical trial used a dose of 1000 mg/day (~14 mg/kg), at the lower end of this scaled range. Advances in oral formulations of vitexin and isovitexin, including chitosan–alginate microencapsulation, β-cyclodextrin inclusion complexes and nanoparticle carriers, have shown promise for improving absorption in preclinical systems [78], with encapsulated formulations reportedly increasing peak plasma concentrations several-fold in rabbits, although these strategies have not yet been evaluated in humans pharmacokinetic and dose-finding studies therefore remain a critical prerequisite for designing adequately powered efficacy trials in patients with established T2DM.

### 7.2. Limitations of the Review

Despite this body of preclinical work, several limitations of the underlying evidence base should be acknowledged. Only one clinical trial has been reported to date, conducted in participants with prediabetes rather than established T2DM; the extract used was not standardised to a defined marker compound, and no human pharmacokinetic data are available for vitexin, isovitexin or the triterpene fraction of *F. deltoidea*. Most in vivo studies employed relatively short treatment durations of 2–8 weeks, and long-term safety, reproductive toxicity and drug–herb interactions have not been systematically evaluated. The majority of preclinical models used streptozotocin-induced diabetes, which more closely resembles type 1 than type 2 diabetes and does not fully reflect the pathophysiology in which *F. deltoidea* is traditionally used. Direct comparisons across studies are further constrained by heterogeneity in plant part, variety, extraction solvent, dose and animal model.

This narrative review itself has additional limitations. Formal risk-of-bias appraisal and quality assessment of the included studies were not performed, and findings were not synthesised through meta-analysis. The literature search was restricted to English-language publications and may have missed relevant studies published in Bahasa Malaysia, Bahasa Indonesia or other regional languages of the areas in which *F. deltoidea* has been most extensively investigated.

### 7.3. Future Research Directions

Several priority areas emerge from the foregoing analysis. First, a standardised *F. deltoidea* extract with defined vitexin, isovitexin and, where feasible, triterpene content should be developed to enable direct comparison across studies and to support regulatory submissions. Second, human pharmacokinetic and bioavailability studies of vitexin and isovitexin are needed, including the effects of formulation on oral absorption and inter-individual variability in metabolism. Third, adequately powered randomised clinical trials in patients with established T2DM should be conducted using a standardised extract, a treatment duration of at least 12 weeks and glycated haemoglobin as the primary endpoint. Fourth, mechanistic and head-to-head varietal studies are needed to clarify the individual and combined contributions of vitexin, isovitexin, triterpenes and phenolic acids to the observed pharmacological activity. Until such evidence is available, *F. deltoidea* is best regarded as a promising botanical candidate that warrants further clinical investigation rather than an established treatment for DM.

## 8. Conclusions

The available preclinical evidence indicates that *F. deltoidea* possesses antidiabetic activity mediated by several complementary mechanisms, including inhibition of α-glucosidase and α-amylase, stimulation of insulin secretion through both K^+^-ATP-dependent and -independent pathways, enhancement of glucose uptake in hepatocytes and adipocytes, stimulation of adiponectin secretion and inhibition of PTP1B. In vivo studies in STZ-induced T1DM and STZ-NAM-induced T2DM models support these observations and additionally show modulation of hepatic gluconeogenic and glucose-metabolic genes, restoration of pancreatic islet architecture and protection against diabetic nephropathy and bone loss. Vitexin and isovitexin are consistently identified as the major bioactive flavonoid C-glycosides, but compounds such as the oleanane-type triterpene 3β,11β-dihydroxyolean-12-en-23-oic acid and other polyphenols also appear to contribute to the activity of the extract. Inter-varietal differences in chemical composition and pharmacological activity, particularly the K^+^-ATP-independent insulin secretion observed with *var. trengganuensis* and the pronounced glucose-lowering effect of *var. intermedia*, suggest that variety standardisation may be important for achieving reproducible activity.

## Figures and Tables

**Table 1:** Summary of in vitro studies on *Ficus deltoidea*.

Model/Assay	Extract/Compound	Main Findings	References
α-glucosidase & α-amylase assays	Fruit aqueous & ethyl acetate fractions	Dose-dependent α-glucosidase inhibition; little effect on α-amylase.	[27]
α-amylase assay; molecular docking	Leaf 50% ethanol-water extract	Concentration-dependent α-amylase inhibition; docking suggests vitexin and isovitexin bind to active site.	[28]
BRIN-BD11 β-cells; 3T3-F442A adipocytes	Aqueous, ethanolic, methanolic extracts (10–1000 μg/mL)	Dose-dependent insulin secretion via K^+^-ATP-dependent and -independent pathways; ↑ glucose uptake; ↑ adiponectin secretion.	[31]
BRIN-BD11 β-cells; seven varieties	Standardised methanolic extracts (10–1000 μg/mL)	All varieties ↑ insulin secretion; only var. trengganuensis acted via K^+^-ATP-independent pathway.	[18]
Chang liver cells	Multiple extracts and fractions (50–500 μg/mL)	Most fractions ↑ basal and insulin-stimulated glucose uptake; insulin-mimetic and -sensitising activity.	[36]
PTP1B inhibition assay	70% ethanol extract; triterpene fraction	~92% PTP1B inhibition at 200 μg/mL; identification of 3β,11β-dihydroxyolean-12-en-23-oic acid.	[25]
Human coronary artery endothelial cells	Leaf extract	↓ TNF-α-induced ICAM-1 and VCAM-1; ↑ eNOS expression.	[50]
Antioxidant assays (TPC, DPPH, CUPRAC, lipid peroxidation); human LDL oxidation (CD, TBARS); HMG-CoA reductase assay; HepG2 cells with palmitic acid-induced lipid accumulation	Aqueous crude extract (CE), water fraction (WF), ethyl acetate fraction (EAF)	CE most active: highest TPC, ↑ DPPH scavenging (IC50 5.47 μg/mL), 76% lipid peroxidation inhibition, ↓ LDL CD and TBARS, ~88% HMG-CoA reductase inhibition, dose-dependent ↓ in PA-induced HepG2 lipid accumulation.	[51]
WRL68 human liver cells; palmitic acid-induced steatosis	Aqueous extracts and ethyl acetate fractions of three varieties	Only var. kunstleri reduced lipid accumulation at 200 μg/mL; altered ubiquitin-proteasome and oxidative stress-related protein expression.	[59]
BV2 microglial cells; LPS challenge	Methanolic leaf extract	↓ TNF-α, IL-1β, IL-6, iNOS, COX-2 and PGE2; suppression of NF-κB pathway.	[55]
Ames test (*S. typhimurium* TA 98, TA 100); V79 cells (menadione-induced oxidative stress); FRAP assay	Aqueous extract of var. deltoidea	Non-mutagenic up to 50 mg/mL; antimutagenic against 2-aminoanthracene; cytoprotective against oxidative stress; concentration-dependent antioxidant capacity.	[58]

↑ indicates increase; ↓ indicates decrease.

**Table 2:** Summary of in vivo studies on *Ficus deltoidea*.

Model/Duration	Extract/Dose	Main Findings	References
ICR mice; OGTT; 4–6 h	Aqueous extract; 50, 100, 200 mg/kg	↓ Peak blood glucose at 180 min; non-toxic in brine shrimp assay.	[60]
STZ-induced T1DM rats; 4–6 h	Ethanolic extract; 100, 500, 1000 mg/kg	↓ Fasting and postprandial hyperglycaemia; ↑ glucose tolerance at 100 mg/kg.	[61]
Normoglycaemic mice and STZ T1DM rats; 14 d/4 wk	Methanol and butanol extracts; 100–400 mg/kg	Both extracts ↓ blood glucose in normoglycaemic mice; only methanol extract effective in diabetic rats; no toxicity at 200 mg/kg.	[62]
Normoglycaemic rats; five varieties	Standardised extracts; 250 and 500 mg/kg	*var. trengganuensis* (250 mg/kg) and *var. intermedia* (500 mg/kg) most effective; LD_50_ > 2000 mg/kg for all varieties.	[22]
STZ T1DM rats; 14 d	Standardised methanolic extract 1000 mg/kg; vitexin and isovitexin 250 and 500 mg/kg	↓ FBG; ↑ insulin; ↑ glucose tolerance; ↓ PEPCK and G6Pase; ↑ GK and PPARγ in liver; ↑ GLUT4 in skeletal muscle.	[21]
STZ-NAM T2DM rats; 28 d	70% ethanol extract; 125, 250, 500 mg/kg vs. metformin 150 mg/kg	↓ FBG; ↑ insulin; improved lipid profile; ↑ antioxidant enzymes; ↓ hepatic *PTP1B*, *PEPCK*, *G6Pase*; ↑ *Slc2a2* and insulin receptor mRNA.	[25]
STZ T1DM rats; 8 wk	Methanolic extract 1000 mg/kg; vitexin	↓ FBG; restoration of pancreatic islet architecture; ↑ SOD and GPx; ↓ lipid peroxidation; ↑ insulin secretion.	[68]
STZ-induced diabetic nephropathy in rats; 8 wk	Standardised methanolic extract; 1000 mg/kg	↓ Serum creatinine, BUN, uric acid; preserved glomerular architecture; ↓ caspase-3 and cytochrome c; ↑ renal SOD, CAT, GPx.	[69]
STZ-induced diabetic rats; 8 wk	Standardised methanolic extract	↑ Bone mineral density and osteocalcin; improved bone microarchitecture.	[70]

↑ indicates increase; ↓ indicates decrease.

**Table 3:** Summary of the clinical trial on *Ficus deltoidea*.

Design and Participants	Intervention	Main Findings	References
8-week, prospective, randomised, double-blind trial; 30 adults with prediabetes (FBG 100–125 mg/dL)	*F. deltoidea* leaf extract 1000 mg/day	↓ Total cholesterol and LDL; no significant change in FBG or fasting plasma insulin; small non-significant ↑ in body weight; no serious adverse events.	[71]

↑ indicates increase; ↓ indicates decrease.

## Data Availability

No new data were created or analysed in this study. Data sharing is not applicable to this article.
